# A Comprehensive Numerical Model for Reservoir-Induced Earthquake Risk Assessment

**DOI:** 10.3390/e25101383

**Published:** 2023-09-26

**Authors:** Xuefeng Peng, Rong Zhao, Kai Deng

**Affiliations:** 1 Key Laboratory of Earth Exploration and Information Technology of Ministry of Education, Chengdu University of Technology, Chengdu 610059, China; pengxf1999@outlook.com (X.P.); rongzhao1124@outlook.com (R.Z.); 2 College of Geophysics, Chengdu University of Technology, Chengdu 610059, China

**Keywords:** reservoir-induced seismicity, Coulomb failure criterion, seismic hazard assessment

## Abstract

The assessment of seismic risk and the prevention of earthquake occurrences during reservoir operation present significant challenges in terms of accurate determination. This study aims to address this issue by developing a numerical model. The primary objective is to estimate the vulnerability of different fault types to reservoir impoundment. This model integrates essential parameters such as fault dip and the relative orientation between the reservoir and potential earthquakes, and it is structured within a risk framework using polar coordinates. Through comprehensive computations, we evaluate the alterations in elastic stress and fluid pore pressure resulting from water impoundment. This is achieved by employing a fully coupled two-dimensional poroelastic approach. Furthermore, our model incorporates relevant seismic data to enhance its accuracy. The findings of our study underscore that the critical factor lies in the fault’s precise positioning with respect to the reservoir. The risk associated with a fault is contingent upon both its location and its orientation, emphasizing the importance of these factors in determining hazardous zones.

## 1. Introduction

The impoundment of artificial reservoirs can trigger earthquakes, leading to loss of life and property, and giving rise to safety issues such as chemical spills [1,2,3,4,5]. This issue has garnered significant attention, particularly with the escalating development of water conservation and hydropower projects. Reservoir impoundment promotes earthquakes by increasing the crustal pore pressure and changes the stress state on pre-existing faults [6,7,8]. Research has predominantly focused on elucidating the diffusion effects of pore pressure [9,10]. For instance, the seismic activity near the Song Tranh 2 Reservoir in Vietnam has been attributed to pore pressure diffusion, observed through time delays subsequent to water impoundment [11]. Similarly, the spatiotemporal seismic trends near the Açu Dam in Northeast Brazil underscore the significance of pore pressure in reservoir-induced seismicity [12]. Exploring the Polyphyto Dam in Northern Greece, Michas et al. [13] indicated a delayed regional seismic response, correlating it with pore pressure diffusion.

Studies have also attempted to synthesize the influences of both diffusion and loading effects. These endeavors often involve the construction of numerical models to elucidate induced seismicity patterns. Notably, the Aswan reservoir-triggered M_L_ 5.7 earthquake in 1981 was assessed through finite element modeling, demonstrating increased pore pressure and Coulomb failure stress [14]. It is under debate whether the May 2008 Ms 8.0 Wenchuan earthquake and the abnormal seismicity prior to it were caused by the impoundment of the nearby Zipingpu Reservoir [15,16,17,18,19]. Tao et al. [20] employed a three-dimensional numerical model to simulate the pore pressure and stress, proposing that the Zipingpu Reservoir’s impoundment in China altered the regional tectonic stress field before the Wenchuan earthquake, although others think the link between the Zipingpu Reservoir and the Wenchuan earthquake is low [18]. Similarly, seismicity around China’s Dagangshan Reservoir was attributed to the combined effects of pore pressure and elastic loading in numerical simulations [21].

Recent studies have highlighted the role of elastic stress perturbation in various induced seismicity scenarios, including reservoir impoundment, fluid injection, mining, and geothermal production [4,22]. Instances such as earthquake sequences in Cushing, Oklahoma and Crooked Lake, Alberta underscore that elastic responses may dominate induced seismicity after fluid injection [22,23]. The extensive seismic events following enhanced geothermal system activities in Basel, Switzerland were attributed to modifications in the ambient stress field due to fluid injection [24]. Recently, earthquake nowcasting [25,26,27], which is based on natural time [28,29], has been applied to induced seismicity by Luginbuhl et al. [26], with very interesting results.

Small–large-magnitude induced earthquakes have prompted the development, in the research community, of seismic risk models that serve both to estimate the impact of these events and to explore the efficacy of different risk mitigation strategies [30,31]. While discussions surrounding elastic stress’s effects on induced seismicity are plentiful, few studies have managed to predict seismic events preemptively, offer reservoir siting guidelines, or delineate risk zones during impoundment. Therefore, the imperative lies in establishing risk assessment models to monitor induced seismicity. This paper addresses this gap by crafting seismic risk maps for earthquake risk mitigation. We investigate the seismic reaction to water impoundment in a mechanically and hydraulically homogeneous medium. Employing a fully coupled poroelastic model, we compute elastic stress and fluid pore pressure arising from surface water impoundment. Our model accounts for fault characteristics and seismic tendencies, substantiated by existing data. Notably, our findings emphasize that earthquakes are prone to occur when faults possess a suitable dip and are strategically located in relation to a reservoir. Ultimately, this model holds promise for enhancing seismic risk management around water reservoirs.

## 2. Materials and Methods

### 2.1. Poroelastic Model

We follow the linear poroelasticity theory [32,33,34] to calculate the stress and pore pressure perturbations resulting from reservoir impoundment. The governing equations of linear poroelasticity for an isotropic, homogeneous medium can be written as follows:

(1) $$\begin{array}{c} G\nabla^{2}u+\frac{G}{1-2\nu }\nabla \epsilon -\alpha \nabla \;p=0, \end{array}$$

(2) $$\begin{array}{c} \frac{1}{M}\frac{\partial p}{\partial t}+\alpha \;\frac{\partial \epsilon }{\partial t}-\nabla \cdot \left(\frac{\kappa }{\eta }\nabla p\right)=0, \end{array}$$

where we have ignored the body force and fluid source for the boundary value problem. In the equations, G is the shear modulus, u is the displacement vector, ν is the Poisson’s ratio under drained conditions, ϵ=∇·u is the volumetric strain, α is the Biot–Willis coefficient, p is the excess pore pressure, M is the Biot modulus, κ is the permeability, and η is the fluid viscosity.

The stress tensor is calculated from the strain and pore pressure through the constitution equation in poroelasticity:

(3) $$\begin{array}{c} \sigma_{ij}=\frac{2G\nu }{1-2\nu }\epsilon \delta_{ij}+2G\varepsilon_{ij}-\alpha p\delta_{ij}. \end{array}$$

In Equation (2), $M^{-1}$ represents the bulk compressibility, which can be measured by evaluating the amount of water in a soil with constant volume under pressure [32], given by

(4) $$\begin{array}{c} M^{-1}=\frac{9}{2}\frac{\left(1-2\nu_{u}\right)\left(\nu_{u}-\nu \right)}{\left(1-2\nu \right){\left(1+\nu_{u}\right)}^{2}GB^{2}}, \end{array}$$

where $\nu_{u}$ is the undrained Poisson’s ratio [33] and B is the Skempton’s coefficient, which quantifies the change in pore pressure against confining pressure [33,35]. The undrained conditions prevail if pore fluid is prevented from escaping or entering [8].

**Figure 1:**
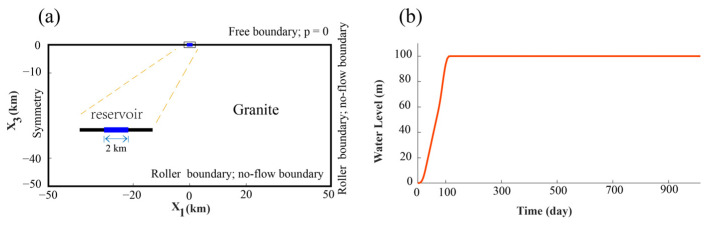
Model configuration: (**a**) The two-dimensional domain is described by a homogeneous granite medium, and the formation properties are given in Table 1. The reservoir is located at the origin of the coordinates. (**b**) The filling history. The water level increases to its maximum at 100 m in 100 days and remains constant.

We began by considering a designated water storage rate applied to a surface area of 2 km (L = 2 km), within a homogeneous full space, as depicted in Figure 1. The reservoir, structured as a two-dimensional entity, spanned 2 km in length and 100 m in depth. Our approach effectively approximated the reservoir as a surface load, while fully accounting for the dynamic history of water filling.

To ensure the fidelity of our simulations, we established a model domain measuring 100 km × 50 km. This choice of dimensions effectively eliminated potential boundary effects. Employing a finite element method (FEM), we solved the two-dimensional boundary value problem. Specifically, our model domain was discretized into 5000 rectangular elements. This considerable scale ensured that our model not only accommodated the necessary breadth but also featured a highly refined mesh. This refinement was essential for effectively capturing boundary effects and addressing gradients that might arise. We defined the bottom and side boundaries to be unrestricted in their sliding motion. Simultaneously, the upper surface assumed the role of a free surface. Furthermore, the seepage gradient at both the bottom and the sides was established at zero. This configuration was designed to establish initial hydrostatic equilibrium within the model.

Table 1 lists the nominal material parameters used in the calculations. We used an average Skempton’s coefficient of 0.75 [36]. The permeability in the reference model corresponded to a diffusivity of 0.42 m^2^/s, which satisfied the laboratory measurements [10].

### 2.2. Coulomb Stress and Seismic Risk Model

We used the Coulomb failure criterion [37] to characterize the tendency of frictional slip. The change in Coulomb failure stress (CFS) is expressed as follows:

(5) $$\begin{array}{c} \Delta CFS=\Delta \tau +\mu \left(\Delta \sigma +\Delta p\right), \end{array}$$

where Δτ is the shear stress change along the slip direction, Δσ is the normal stress change acting on the fault plane (positive for tension), Δp is the excess pore pressure, and μ is the coefficient of friction. A positive ΔCFS promotes slip, while a negative one prohibits slip.

For an arbitrary point (x_1_, x_3_) in the model domain, we calculated the Coulomb stress for normal or thrust faults with arbitrary dip angles. On this basis, we were able to find the faults that were more likely to be activated. Combing the results at all points, we provided a spatial distribution of the seismic risk.

**Figure 2:**
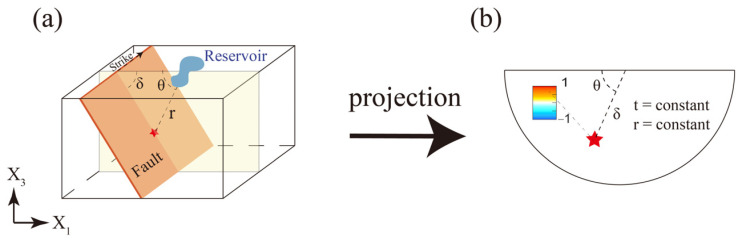
The projection method: (**a**) A schematic showing the location of a fault relative to the reservoir. The fault is represented by an orange plane, the red star indicates the hypocenter, the blue area represents the reservoir, and the yellow plane represents the vertical cross-section containing the geometric center of the reservoir and the hypocenter. The strike of the fault is perpendicular to the cross-section. The dip angle and polar angle are denoted by δ and θ, respectively. (**b**) Projection of the hypocenter onto the θ−δ plane. The seismic risk map shows normalized Coulomb stress for a constant distance at any time after water impoundment.

We assumed a uniform distribution of faults in the medium, and the location a possible hypocenter can be characterized by its distance from the geometric center of the reservoir $r=\sqrt{x_{1}^{2}+x_{3}^{2}}$ and the polar angle $\theta =\arccos\left({-x}_{1}/r\right)$, where θ is a counterclockwise rotation from the horizontal (Figure 2a).

For a specific distance *r*, we made a seismic risk map by calculating the normalized Coulomb stress change for every polar angle and dip angle. In the schematic risk map, a fault with a polar angle θ and a dip angle δ is represented by a point $\left(\theta ,\;\delta \right)$ in the θ−δ plane. Here, the radius in the plot represents the dip angle (Figure 2b). We calculated the Coulomb stress change at every point in the polar plane and normalized the values against the maximum Coulomb stress change. The normalized $\Delta CFS\left(\theta ,\delta \right)$ is presented by color on the seismic risk map, where warm and cold colors indicate high and low seismic risk, respectively.

## 3. Results

### 3.1. Numerical Results

**Figure 3:**
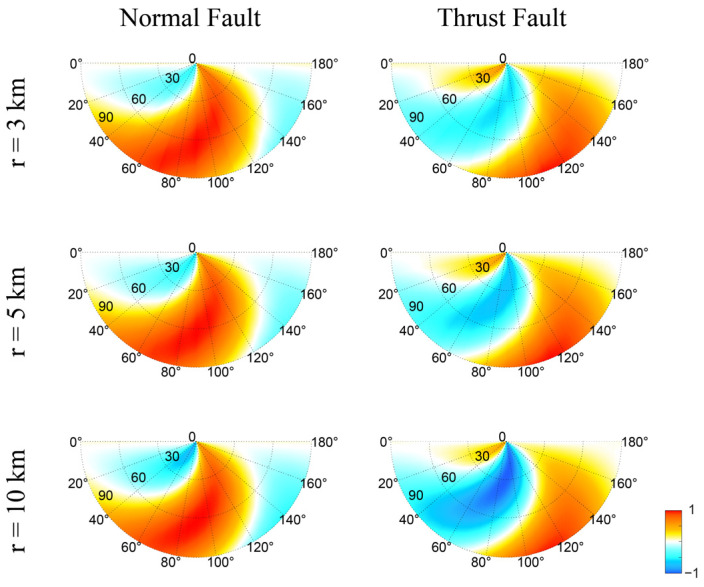
Snapshots of normalized Coulomb failure stress for normal and thrust faults at r=3, 5, and 10 km at t=1000 days after reservoir impoundment.

Figure 3 shows the seismic risk maps for normal and thrust faults for different distances (3, 5, and 10 km). For normal faults, the high-risk area is mostly right beneath the reservoir (polar angle 60~120°). Those faults with dip angles of 50~80° are most susceptible to reservoir loads. The pattern of the seismic risk does not change significantly when the distance r changes. On the contrary, thrust faults located beneath the reservoir will be stabilized under the loads. Although the seismic risk generally reduces at greater distances, the overall distribution of positive and negative Coulomb stress does not change with distance.

**Figure 4:**
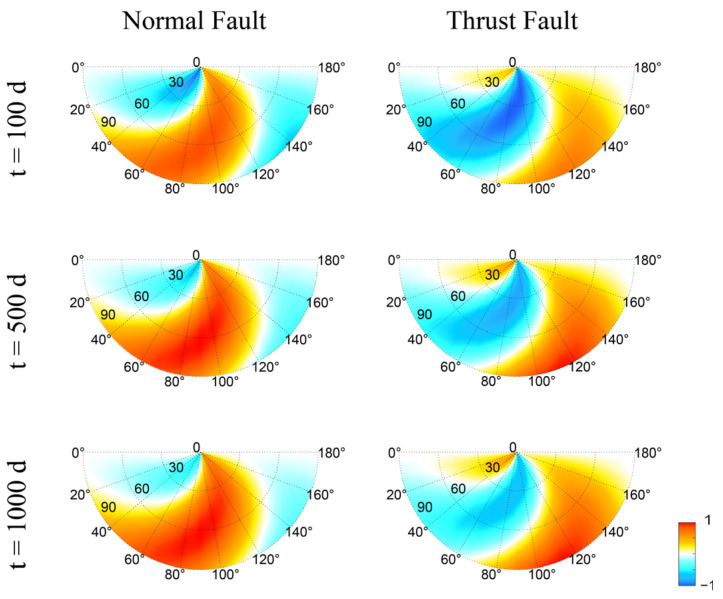
Snapshots of normalized Coulomb failure stress for normal and thrust faults at t=100, 500, and 1000 days after reservoir impoundment for r=5 km.

Figure 4 shows the seismic risk maps for normal and thrust faults at different times after the water impoundment for r=5 km. Due to the delayed increase in pore pressure, the seismic risk increases with time, especially for those areas that are at low risk initially. However, the general pattern (i.e., the distribution of areas at risk) does not change significantly with time. Figure 3 and Figure 4 suggest that we can use the seismic risk map made at a moderate distance and time to conduct risk analysis for reservoir-induced seismicity.

### 3.2. Application to Reservoir-Triggered Earthquakes

**Figure 5:**
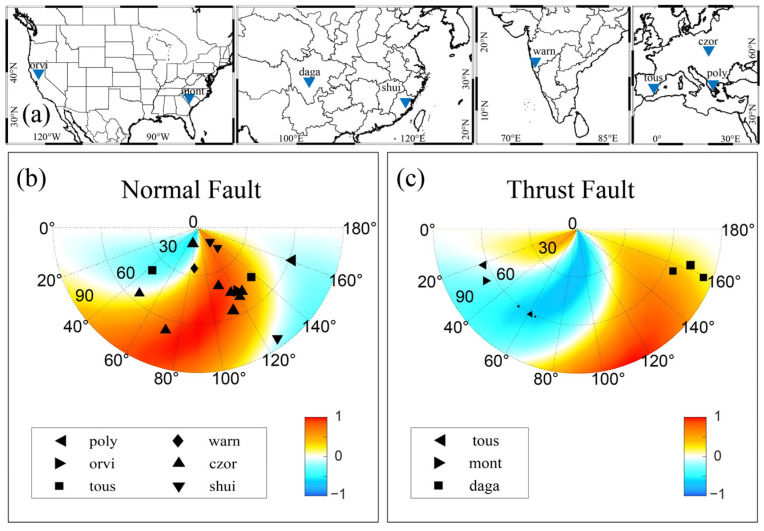
The reservoir-induced earthquakes projected onto the risk model: (**a**) Maps showing the locations of the reservoirs. (**b**,**c**) The normal and thrust earthquakes projected onto the risk model, respectively. The Coulomb failure stress was calculated for r=5 km and t=1000 days. Abbreviations: poly = Polyphyto Dam; orvi = Orville Dam; tous = Tous New Dam; warn = Koyna-Warna Dam; czor = Czorsztyn Lake; shui = Shuikou Reservoir.

Well-documented reservoir-triggered earthquakes were projected onto our risk model (Figure 5). The cases of reservoir-induced earthquakes included the Koyna-Warna sequence in India [38], the Czorsztyn M 4.8 earthquake in Poland [39], the Oroville earthquakes (Pacific Earthquake Engineering Research Center (PEER)) and the Monticello earthquake sequence [6] in the United States, the Dagangshan seismic sequence [40] and Shuikou sequence [41] in China, the Tous earthquakes in Spain [42], and the earthquakes near the Polyphyto Dam in Greece [13]. The earthquake catalogs and the fault solutions are included in Table S1. It is accepted that the pore pressure plays a significant role at short distances to the reservoir [17,18]; the proposed projection method is less effective at short distances because the pore pressure dominates over the elastic stresses. Faults of any orientation will be at high risk (Figure S1). We thus ignore the earthquakes that were within 1 km of the reservoir in the application below. About 80% of the earthquakes occurred in areas where the normalized Coulomb stress was positive, and the earthquakes were mainly concentrated on the fault range from 30° to 60° dip angle. The outliers may be related to unknown fluid pathways that are beyond the scope of this study.

## 4. Discussion

The interaction between solid matrix stress and pore pressure variations resulting from reservoir impoundment plays a pivotal role in triggering earthquakes along pre-existing faults. Accurately identifying fault segments at elevated risk of activation stands as a crucial step in effective hazard mitigation strategies. Presently, the primary tool for monitoring revolves around intensive station observation, which is reliant on seismicity and fault slip data. Regrettably, this approach falls short in its predictive capabilities. By conducting comprehensive geological surveys around reservoirs and integrating the earthquake nowcasting [26,27] with the risk model proposed here, it becomes possible to predict the time and location of reservoir-induced earthquakes and substantially mitigate the associated seismic hazard. This study unveils a consistent pattern wherein seismicity escalates beneath the reservoir in the presence of a normal fault with a 30~60° dip. Furthermore, destabilization tendencies emerge in areas marked by a steeply dipping thrust fault, particularly when the reservoir is positioned at the fault’s footwall or on the hanging wall of a gently inclined thrust fault. The methodology presented here holds the promise of serving as a blueprint for a generalized reservoir earthquake model. This approach effectively categorizes fault types that pose heightened risks under reservoir loading conditions. Future extensions of this analysis could encompass multiple fault scenarios, incorporate three-dimensional models, and explore the dynamics of strike-slip faults.

### 4.1. Fault Risk Tendency

The potential of fault triggering is profoundly influenced by the relative positioning of the fault with respect to the reservoir, as well as by the specific geometry of the fault. Utilizing our model, we adeptly delineated zones of heightened risk. Notably, the area of concern encompasses scenarios where a steep normal fault is directly situated beneath a reservoir. The propensity for seismic induction is particularly pronounced when the reservoir is positioned on the footwall of a steeply dipping thrust fault or on the hanging wall of a shallowly dipping one (Figure 5). Roeloffs [7] found that reservoir-induced seismicity’s impact is contingent upon the fault’s characteristics and location, a notion further discussed by Talwani [8], emphasizing the reservoir’s stability in relation to fault dynamics. Our model not only corroborates Roeloffs’ theory but also extends it, providing a more comprehensive and intuitive representation of the phenomenon (Figure 5).

It is important to note that the delineated risk area is not indefinitely hazardous; it is influenced by pore pressure diffusion and temporal decay. Our findings demonstrate that the influence of diffusion intensifies over time (Figure 4), with the rate being permeability-dependent [10,43]. Concurrently, induced seismicity follows a consistent pattern of temporal decay. The poroelastic response resulting from reservoir loading can gradually regress over months or even a few years, eventually reaching pre-event levels, as depicted by seismicity rate models [44,45,46,47]. The seismic activity encircling the reservoir emerges as an intricate interplay of elastic stress, pore pressure dynamics, and temporal decay effects.

### 4.2. Simplified Model Setup

Notably, the faults in this paper were distributed uniformly within the medium, and their elastic and hydraulic properties were consistent with those of the background medium. However, it is important to acknowledge that the permeability of a fault can vary significantly, causing the fault zone to function either as a conduit or as a barrier for fluid flow dynamics [48]. In this study, we deliberately focused on a simplified and homogeneous scenario. This choice serves to underscore the intricate relationship between fault attributes and reservoir locations in the context of earthquake nucleation. To delve into the impact of heterogeneous media incorporating faults, further investigations are warranted.

Our approach employs a two-dimensional model to simulate the alterations in elastic stress and fluid pore pressure caused by water loading. It is worth noting that the two-dimensional model does magnify the calculation results, with values approximately two to four times higher than those of the three-dimensional model [19]. The limitation of the two-dimensional model lies in its consideration of changes in only two dimensions, effectively setting the third dimension to infinity. This inevitably neglects the alterations in the third dimension and accentuates the influence of the two dimensions considered. Of course, it is essential to recognize that the computational demands of a three-dimensional model increase exponentially. The pragmatic utility of the two-dimensional model lies in its ability to quantify results to a reasonable extent, all while significantly conserving computational resources and time investments. As computers’ capabilities continue to evolve, the realm of three-dimensional simulations holds promise for further exploration and research.

## 5. Conclusions

In this study, we conducted a comprehensive analysis of the Coulomb failure stress resulting from poroelastic stress and pore pressure alterations in a two-dimensional framework of linear poroelasticity. The culmination of our findings was projected onto a polar coordinate system, where the rotation angle θ and radius δ offer insightful perspectives. Our results notably reveal a heightened seismic activity when a normal fault exhibits a steeper dip directly beneath the reservoir. Additionally, destabilization tendencies emerge when the reservoir is situated either on the footwall of a sharply inclined reverse fault or on the hanging wall of a shallowly dipping thrust fault. It is imperative to underscore that not all faults encompassing the reservoir exhibit the potential to induce seismicity, as this propensity is profoundly influenced by fault characteristics and the precise reservoir location.

Our study significantly underscores the selective nature of fault-induced seismicity, pointing to the pivotal interplay between fault attributes and reservoir positioning. As such, the implications of our research are poised to provide valuable insights for assessing the risks associated with induced seismicity. In practical terms, any project involving reservoirs should diligently consider the specific fault types at play and meticulously construct a detailed risk model. By doing so, the safety of the surrounding region can be more effectively secured, minimizing potential seismic hazards.

In conclusion, our investigation contributes a nuanced understanding of the intricate relationships between fault mechanics, reservoir placement, and induced seismicity. As we move forward, the lessons gleaned from this study have the potential to substantially enhance risk assessment protocols and safeguard against the adverse effects of reservoir-induced seismic activity.

## Figures and Tables

**Table 1:** Parameters for the reference model.

Model Parameter	Symbol	Value
Young’s modulus	E	37.5 GPa
Drained Poisson’s ratio	ν	0.25
Undrained Poisson’s ratio	$\nu_{u}$	0.34
Skempton’s coefficient	B	0.75
Fluid viscosity	η	1 × 10^−3^ Pa·s
Permeability	κ	2.5 × 10^−18^ m^2^
Diffusivity	*c*	0.42 m^2^/s

## Data Availability

The seismic data are publicly available. The numerical data are available upon request to the authors.

## Supplementary Materials

The following supporting information can be downloaded at: https://www.mdpi.com/article/10.3390/e25101383/s1, Figure S1: Projection of earthquakes onto seismic risk maps at different distances; Table S1: The earthquake catalogs and the fault solutions.
