# Supplementary material for: A Comprehensive Numerical Model for Reservoir-Induced Earthquake Risk Assessment
Source: Entropy (Basel). 2023 Sep 26;25(10):1383. doi: 10.3390/e25101383 (PMC10606261; doi:10.3390/e25101383)
Supplement: Supplementary file 1 [file entropy-25-01383-s001.zip › Figure_S1.pdf]

entropy

MDPI

Supplement Material

Figure S1

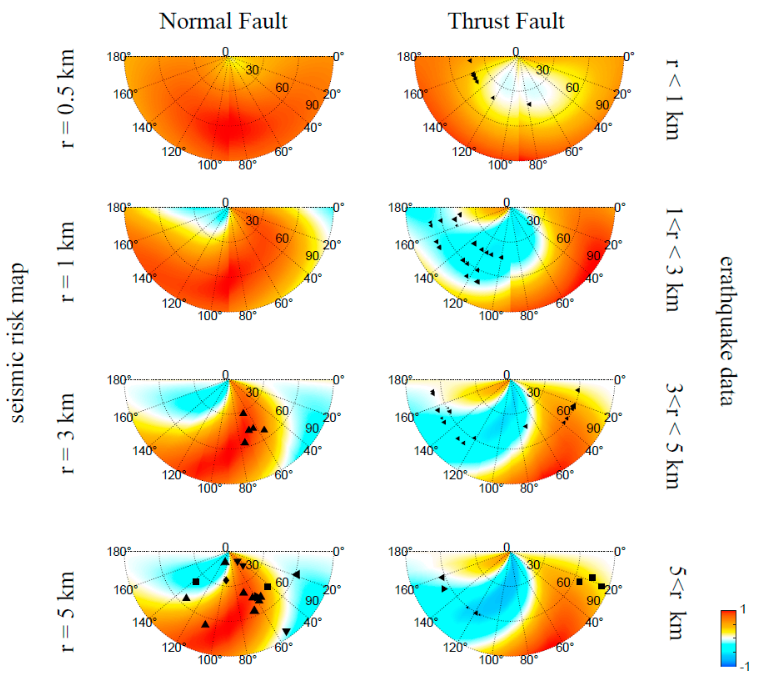

Figure S1. Projection of earthquakes onto seismic risk map at different distances. The background maps are from snapshots of normalized coulomb failure stress for normal and thrust faults at $r = 0.5, 1, 3,$ and $5\ km$ when $t = 1000\ day$ after reservoir impoundment. The earthquake data from Table S1, the data are divided into four groups with the distance to the reservoir, and the legend inherited Figure 5.
